# Smokers’ Likelihood to Engage With Information and Misinformation on Twitter About the Relative Harms of e-Cigarette Use: Results From a Randomized Controlled Trial

**DOI:** 10.2196/27183

**Published:** 2021-12-21

**Authors:** Jessica Liu, Caroline Wright, Philippa Williams, Olga Elizarova, Jennifer Dahne, Jiang Bian, Yunpeng Zhao, Andy S L Tan

**Affiliations:** 1 Department of Social and Behavioral Sciences Harvard TH Chan School of Public Health Boston, MA United States; 2 Population Health Sciences Bristol Medical School University of Bristol Bristol United Kingdom; 3 Play Collaborate Change Boston, MA United States; 4 Department of Psychiatry and Behavioral Sciences College of Medicine Medical University of South Carolina Charleston, SC United States; 5 Department of Health Outcomes & Biomedical Informatics College of Medicine University of Florida Gainesville, FL United States; 6 Annenberg School for Communication University of Pennsylvania Philadelphia, PA United States

**Keywords:** e-cigarettes, misinformation, Twitter, social media

## Abstract

**Background:**

Information and misinformation on the internet about e-cigarette harms may increase smokers’ misperceptions of e-cigarettes. There is limited research on smokers’ engagement with information and misinformation about e-cigarettes on social media.

**Objective:**

This study assessed smokers’ likelihood to engage with—defined as replying, retweeting, liking, and sharing—tweets that contain information and misinformation and uncertainty about the harms of e-cigarettes.

**Methods:**

We conducted a web-based randomized controlled trial among 2400 UK and US adult smokers who did not vape in the past 30 days. Participants were randomly assigned to view four tweets in one of four conditions: (1) e-cigarettes are as harmful or more harmful than smoking, (2) e-cigarettes are completely harmless, (3) uncertainty about e-cigarette harms, or (4) control (physical activity). The outcome measure was participants’ likelihood of engaging with tweets, which comprised the sum of whether they would reply, retweet, like, and share each tweet. We fitted Poisson regression models to predict the likelihood of engagement with tweets among 974 Twitter users and 1287 non-Twitter social media users, adjusting for covariates and stratified by UK and US participants.

**Results:**

Among Twitter users, participants were more likely to engage with tweets in condition 1 (e-cigarettes are as harmful or more harmful than smoking) than in condition 2 (e-cigarettes are completely harmless). Among other social media users, participants were more likely to likely to engage with tweets in condition 1 than in conditions 2 and 3 (e-cigarettes are completely harmless and uncertainty about e-cigarette harms).

**Conclusions:**

Tweets stating information and misinformation that e-cigarettes were as harmful or more harmful than smoking regular cigarettes may receive higher engagement than tweets indicating e-cigarettes were completely harmless.

**Trial Registration:**

International Standard Randomized Controlled Trial Number (ISRCTN) 16082420; https://doi.org/10.1186/ISRCTN16082420

## Introduction

e-Cigarette use is associated with potentially health risks owing to exposure to particulate matter, metals, and other constituents [1]. However, there is growing evidence that the short-term health risks of vaping nicotine are considerably lower than smoking regular cigarettes [1,2]. Recent studies among current smokers reported misperceptions that e-cigarettes are as harmful or more harmful than smoking are increasing in both the United Kingdom and the United States [3]. Misperceptions about the relative harms of e-cigarettes compared with smoking may deter smokers from considering switching to e-cigarettes to reduce their harm from continuing to smoke combustible cigarettes [4,5].

While recent research has described e-cigarette marketing and information on various social media platforms [6-10], there is limited knowledge on the types and sources of e-cigarette–related information and misinformation on social media and how such information and misinformation influences public misperceptions about e-cigarette harms. Misinformation can be defined as information that is incorrect or misleading [11], which differs from misperceptions, defined as false or inaccurate beliefs of the individual. Some examples of misinformation about e-cigarettes include e-cigarettes as being as or more harmful than combustible cigarettes, or that e-cigarettes are completely harmless. Specifically, there is a knowledge gap in assessing smokers’ engagement with information and misinformation about the relative harms of e-cigarettes compared with smoking. Measuring audiences’ engagement with health information and misinformation on social media, such as Twitter, can provide important insights as to how misinformation spreads and potentially impact users’ vaping behavior. The theory of planned behavior posits that intentions are strong predictors of behavior [12]; thus, the likelihood of engagement can be a predictor for actual engagement with information and misinformation.

Moreover, research shows that health rumors and health information and misinformation can undermine public health efforts because misinformation is disseminated more quickly and widely than accurate information on the internet [13]. Perceived message importance can mediate the sharing of information and misinformation on the internet [13]. There have been some studies on engagement done on other platforms but not many focus on Twitter, which is a popular social media platform that many people frequent to discover news and information. Other studies that explore information and misinformation data on Twitter are more descriptive or focus on the content of Twitter posts [14-16], or look at tobacco use as the outcome rather than engagement as the outcome [17]. There is a need for more scientific evidence looking at engagement with misinformation on social media to better develop public health interventions [18].

To address this research gap, we analyzed data from a larger web-based randomized controlled experiment to compare smokers’ likelihood to engage with various forms of information and misinformation on Twitter related to e-cigarette harms. We looked at and compared the United States and the United Kingdom specifically, since regulations and public perceptions of e-cigarettes differ in these 2 countries, and we wanted to examine the relationships across these contexts. Information and misinformation about e-cigarettes on social media are prevalent, and this exploratory study on one social media platform, Twitter, helps examine whether exposure to information and misinformation about e-cigarettes impacts the likelihood of engagement. These findings will inform future work to replicate studies across additional social media platforms and research to measure actual engagement with information and misinformation.

## Methods

### Methods Overview

**Figure 1:**
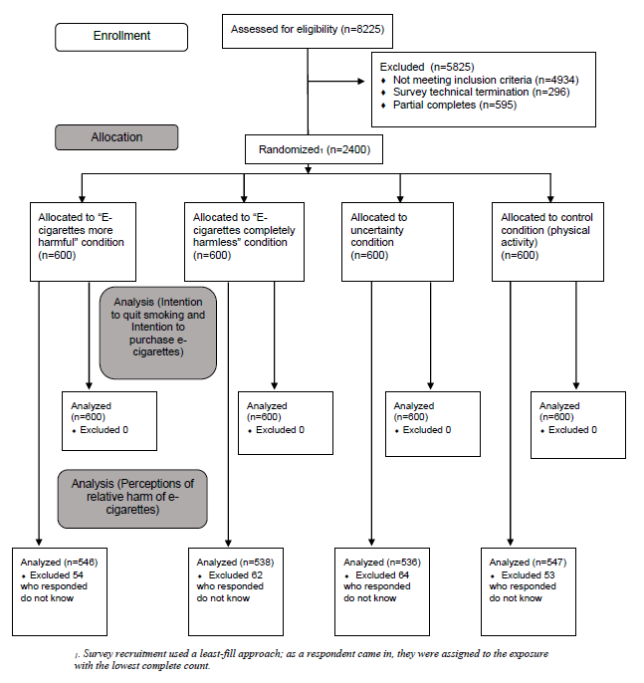
CONSORT (Consolidated Standards of Reporting Trials) flow diagram.

Data for this analysis was obtained from a web-based experiment among 1200 US and 1200 UK adult smokers. The study’s primary objective was to examine the effects of exposure to information and misinformation on e-cigarette harms on Twitter on smokers’ intentions to quit smoking and use e-cigarettes [19]. This analysis focuses on the measures of likelihood to engage with misinformation on e-cigarette harms on Twitter, which were collected as part of the overall study. Participants were enrolled through the web-based consumer research panel Prodege, recruited via internet sources, such as email invitations, telephone alerts, banners and messaging on websites, and online communities (CONSORT [Consolidated Standards of Reporting Trials] diagram in Figure 1). Eligible participants were aged 18 years and older, smoked cigarettes in the past 30 days, and had not used e-cigarettes in the past 30 days.

Participants first completed baseline measures of their intentions to quit smoking and use e-cigarettes. Next, participants were randomly assigned through the built-in least-fill randomizer function on the Prodege survey platform to view four tweets within one of the following four experimental conditions in a 1:1:1:1 ratio: (1) e-cigarettes are as harmful or more harmful than smoking, (2) e-cigarettes are completely harmless, (3) uncertainty, or (4) control (physical activity). Based on the current state of the science of e-cigarette harms [1,2], conditions 1 and 2 represented the misinformation tweets, and condition 3 represented comments of media discourse on Twitter often claiming the evidence on e-cigarette harms is uncertain or questioned scientific claims.

The tweets shown to participants were captured through a validated machine learning algorithm developed by the study team [20,21]. We chose to use real tweets rather than artificially created ones to allow for a more realistic representation of what participants would potentially encounter on social media, and this would provide more external validity for the study’s results. Using the random sample function in SPSS, 499 tweets were identified from a larger corpus of over 700,000 tweets about e-cigarette harms, which was then narrowed down to 20 tweets per experimental condition by the study team. Inclusion criteria for the tweets were the following: (1) explicit statement that e-cigarettes were either as or more harmful than smoking, completely harmless, or uncertain; (2) no mention of children or young people; (3) no mention of specific diseases; (4) no profanities; (5) had multiple “likes” or “retweets”; (6) no advertising; (7) no pictures; and (8) was available publicly (ie, not deleted). We then selected four representative tweets for each of the experimental conditions. Tweets for the control condition comprised 4 physical activity tweets to reduce bias and avoid topics related to e-cigarettes and substance use. Within each condition, participants viewed 4 tweets in the same order. Multimedia Appendix 1 displays the tweets that comprised each condition.

In this study, we focused narrowly on the topic of e-cigarette relative harms versus short-term harms of smoking cigarettes and relied on the state of the science that was contemporaneous to the occurrence of tweets and when the study was conducted [1,2]. In the United Kingdom, e-cigarettes are tightly regulated and have been endorsed as a harm reduction strategy for smokers [22]. The conclusions from these reports are reflected in public health agencies’ health messaging in the United States and the United Kingdom that e-cigarettes are 95% less harmful than continuing to smoke cigarettes [23,24]. In addition, the most recent Public Health England report concluded that the relative risk of adverse health effects from e-cigarette use are expected to be substantially lower than conventional cigarette smoking [25]. This provided the rationale for categorizing tweets to the contrary as misinformation in this study. However, we recognized evidence of absolute* *health effects from e-cigarette use and therefore categorized tweets that indicated e-cigarette use being completely harmless as misinformation.

Following each tweet, participants were shown a brief description of what it means to reply, retweet, like, and share a message on Twitter. They were then asked to indicate whether they are likely to reply, retweet, like, or share the tweet they just viewed. Before answering these questions, participants were also provided with a link to Twitter’s official definitions of each form of engagement (Multimedia Appendix 2). They were then asked to complete posttest measures of intentions to quit smoking and use e-cigarettes, followed by questions regarding demographics and tobacco use. Participants were asked how often they visited or used eight different social media platforms (Twitter, Instagram, Facebook, Snapchat, YouTube, WhatsApp, Pinterest, and LinkedIn) on a 6-point scale ranging from several times a day to never. We ran a randomization check and confirmed randomization was successful because baseline characteristics did not differ across the 4 conditions. The University of Bristol’s institutional review board approved this study.

### Measures

#### Outcome Measure: Likelihood of Engagement With Tweets

We operationalized the likelihood of engagement with tweets as the likelihood of replying, liking, retweeting, or sharing such information based on prior research on engagement with news and health information on Twitter [26]. These forms of engagement represent the 4 options that Twitter users can choose to interact with every tweet within the Twitter platform. After reading each of the 4 tweets in their assigned experimental condition, participants were asked 4 questions, which included “Are you likely to Reply/Retweet/Like/Share this message?” Response options were “yes (1)” or “no (0).” Multimedia Appendix 3 summarizes the mean (SD) values of the four distinct engagement variables by condition. The Kuder-Richardson coefficient (KR-20) across the 16 engagement items was 0.93, indicating high internal consistency. We created a combined likelihood of engagement index by summing the responses to the 16 engagement questions (range 0-16).

#### Covariates

We obtained participants’ characteristics including sex (male or female), country (the United Kingdom or the United States), race (White or non-White), education (high/secondary school or below; some college/further education college; college/university degree or higher), age (in years), social media use (daily use of 8 different social media platforms; eg, Twitter, Facebook, Instagram, and YouTube), daily internet use (hours per day), past e-cigarette use (never or ever), and baseline perceived relative harm of e-cigarettes compared to combustible cigarettes (Likert scale of much less harmful to much more harmful).

### Statistical Analysis

The analytic sample comprised participants who reported using any of the social media platforms at least once a month—974 used Twitter at least once a month and 1287 participants never used Twitter but had used other social media platforms at least once a month. We excluded 139 participants who reported that they never used any of the 8 social media platforms as the questions on likelihood of engagement (reply, retweet, like, and share a tweet) may not be meaningful for these participants. Although the experimental stimuli were presented in the specific context of a tweet, we included both Twitter users and those who used other social media in our analysis because we expected that those who used other social media would be familiar with the concept of engaging with tweets.

We used the R software for coding and analysis of the data. We first conducted descriptive analyses of the individual likelihood of engagement variables (reply, retweet, like, and share) and the combined likelihood of engagement measure stratified by condition. Next, we performed a bivariate Poisson regression of likelihood of engagement as the outcome, treating the overall combined engagement variable as a positive count variable, and condition as a categorical predictor. Condition 1 (tweets that e-cigarettes are as harmful or more harmful than smoking) was used as the referent condition to allow for comparison to the condition portraying e-cigarettes most negatively. We then fitted a Poisson regression of likelihood of engagement as the outcome, adjusting for covariates among Twitter users and those who used other social media.

We stratified the bivariate and multiple regression models by country to analyze the association between condition and engagement among US and UK samples with Twitter users and other social media users. Nagelkerke *R*^2^ and Akaike information criterion values were calculated for each regression model to determine the overall goodness of fit, accounting for the number of parameters in the model. There were no missing values for the engagement variable as well as covariates. To compare engagement in conditions 1, 2, and 3 versus that in condition 4 (control), we repeated the above analyses using condition 4 as the referent condition for both samples (Multimedia Appendices 4 and 5).

## Results

### Participant Characteristics

Table 1 summarizes the participant characteristics by country among Twitter users and other social media users. Compared to UK participants, US participants tended to be older, more educated, and more racially diverse. We found that the participant characteristics among Twitter and other social media users were similar (refer to Table 1 for more details).

**Figure 2:**
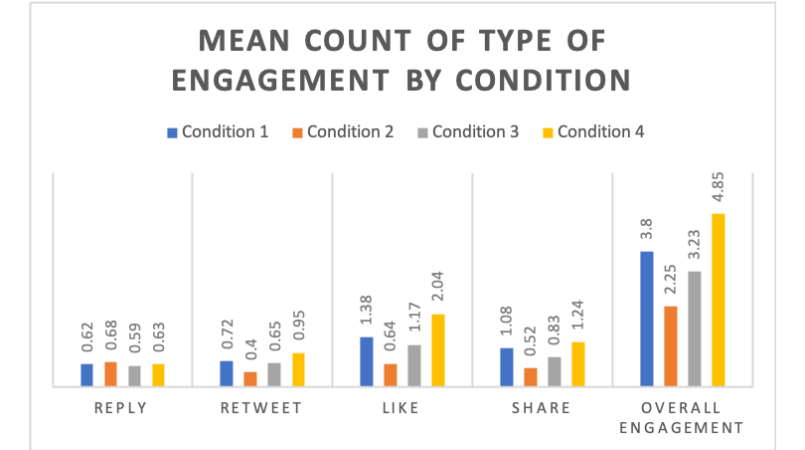
Mean values of the counts of the specific types of engagement as well as overall engagement, by condition.

Figure 2 summarizes the means of the counts of the specific types of engagement as well as overall engagement, by condition.

**Table 1:** Study sample characteristics among Twitter users and other social media users.

Characteristics		Twitter users		Other social media users	
		United States (n=449)	United Kingdom (n=525)	United Kingdom (n=676)	United Kingdom (n=611)
Age (years), mean (SD)		47.7 (13.2)	40.2 (13.4)	50.7 (13.8)	45.3 (14.5)
Females, %		51.0	43.1	51.9	46.0
Non-White, %		32.3	6.9	28.0	6.9
**Education, %**					
	High/secondary school or below	25.2	34.5	34.3	46.8
	Some college/further education college	40.5	36.6	37.7	36.0
	College/university degree or higher	34.3	29.0	28.0	17.2
Never vaped/used an e-cigarette, %		47.7	43.2	48.8	46.8
Social media use, mean (SD; range^a^)		2.6 (1.7; 0-8)	3.0 (1.8; 0-8)	1.4 (1.1; 0-7)	1.7 (1.3; 0-7)
Daily internet use, mean (SD; range)		7.2 (4.8; 0-24)	6.1 (4.1; 0-24)	6.3 (4.4; 0-24)	5.3 (3.6; 0-24)

### Predictors of Likelihood of Engagement Among Twitter Users

Multimedia Appendix 6 summarizes the regression analyses of the association between the condition and the combined engagement measure among Twitter users and stratified by US and UK participants. Among Twitter users, participants were more likely to engage with tweets in condition 1 (e-cigarettes are as harmful or more harmful than smoking) than in condition 2 (e-cigarettes are completely harmless). Across the various models, we found that country, age, race, education, increased social media use, and baseline perceived harm that e-cigarettes are more harmful than combustible cigarettes were associated with increased likelihood of engagement (Multimedia Appendix 6).

These findings were substantively similar in stratified analyses among US and UK participants, except for condition 3 (uncertainty about e-cigarette harms). In the overall sample, participants were not significantly more likely to engage with tweets in condition 1 (e-cigarettes are as harmful or more harmful than smoking) than in condition 3 (uncertainty about e-cigarette harms). However, after stratifying by country, US participants were less likely to engage with tweets in condition 3 than in condition 1, and UK participants were more likely to engage with tweets in condition 3 than in condition 1.

### Predictors of Likelihood of Engagement Among Other Social Media Users

Multimedia Appendix 7 summarizes the regression analyses of the association between condition and the combined engagement measure for the overall study sample and stratified by US and UK participants. In the overall sample of other social media users, participants were more likely to likely to engage with tweets in condition 1 (e-cigarettes are as harmful or more harmful than smoking) than in conditions 2 (e-cigarettes are completely harmless) and 3 (uncertainty about e-cigarette harms). Across the models, country, age, race, education, social media use, and daily internet use were associated with an increased likelihood of engagement (Multimedia Appendix 7). In the UK stratified sample, social media users were not significantly more likely to engage with condition 4 (the control condition) than with condition 1 (e-cigarettes are as harmful or more harmful than smoking).

## Discussion

### Principal Findings

To our knowledge, this is one of the first studies to examine differences between Twitter and social media users’ likelihood of engagement with web-based health-related information among US and UK smokers who are not currently using e-cigarettes. Utilizing a randomized controlled experiment for a web-based sample of US and UK adult smokers, we found that participants were more likely to engage with tweets that stated e-cigarettes were as harmful or more harmful than smoking—specifically retweets, likes, and shares—compared with tweets indicating e-cigarettes were completely harmless. Among Twitter users, there were differences in the US versus the UK sample in the likelihood of engagement with tweets in the uncertainty condition compared with tweets that e-cigarettes were as or more harmful. Although the overall likelihood of engagement was modest across the conditions, these findings indicate meaningful differences between potential engagement with tweets displaying misinformation of e-cigarettes’ relative harm versus smoking and tweets on information and misinformation of e-cigarettes being harmless among smokers.

In the context of increasing trends of misperceptions that e-cigarettes are as harmful or more harmful than smoking among US adult smokers [4], our findings indicate the need for further investigation of public health implications of the increased likelihood of engagement with misinformation that e-cigarettes are as harmful or more harmful as smoking and the underlying reasons. Knowledge of the impact of misinformation is important to inform the development of corrective approaches or media literacy interventions to ensure that smokers have accurate perceptions of the relative harms of e-cigarettes and to help smokers make informed decisions for reducing harm [13,27]. Research is also needed to understand the underlying cognitive and affective mechanisms that motivate smokers’ likelihood to engage with information on social media about e-cigarettes’ relative harm versus smoking. The influence of the internet on population health is continuing to expand, and there is a need to better understand how people are increasingly engaging with “health social media” [28,29]. Prior content analyses of Twitter posts support the importance of incorporating social media into tobacco-related interventions [30,31], and research supports the potential of using Twitter as a means to engage the public in health promotion [32,33].

Our differing findings of Twitter and non-Twitter social media users as it relates to engaging with uncertain information on the internet presents preliminary evidence that we cannot generalize these findings to all social media users. The next steps leading from this research would be to replicate this study to examine information and misinformation about e-cigarette harms, especially in the context of being exposed to uncertain information on other social media platforms, such as Facebook and Instagram, and among users of those specific platforms. Knowledge of the impact of misinformation could also be used to advocate for the use of emerging approaches, such as infodemiology [34-36], to further research the phenomena in the population and to inform public health and public policy. Uncertainty may be perceived differently depending on the social media platform and their users from different countries. However, our mostly similar results comparing Twitter and non-Twitter social media users show how social media users of other platforms can still provide insight into what forms of e-cigarette information are more likely to spread on the internet.

### Limitations

This study was limited by the measurement of participants’ likelihood of engagement with information and misinformation on Twitter in the setting of a web-based study. Study participants were part of an opt-in panel and were not representative of US and UK smokers. In addition, in the interest of presenting actual tweets and not experimentally manipulated tweets, the selected tweets in within the 4 conditions differed in various aspects (eg, names and credentials of the users posting the tweet, length of the tweet, and the number of likes or retweets) in addition to differences in the content regarding e-cigarette harms. The rationale for using actual tweets was to retain the original content of the tweets occurring in the real world. Future research may consider replicating this study using experimentally manipulated tweets to keep other message characteristics constant across conditions. This study did not address nuances in potential absolute harms arising from e-cigarette use, such as youth use and abuse liability, higher dose of nicotine delivery, and individuals creating their own mixtures of e-liquids. Our approach does not address youth e-cigarette use because it was beyond the scope for our initial research questions. We acknowledged the potential harms and therefore categorized tweets that mention e-cigarettes as harmless to be misinformation. Future research is needed to better assess how the public engages with information and misinformation on social media, which describes harms associated with e-cigarette use.

### Conclusions

Despite the limitations, this study provides preliminary evidence that brief exposure to information regarding e-cigarettes being as harmful or more harmful than smoking on social media may be associated with increased levels of engagement compared to information that e-cigarettes are harmless, and this was consistent across Twitter and other social media users and across US and UK participants. But when compared to uncertain information, the pattern of findings was more nuanced and differed between Twitter and other social media users. This requires further investigation, and future research may consider exploring how length of engagement, as well as individual characteristics of the social media post itself such as source of information, content, number of replies, retweets, likes, or shares on the post, and other characteristics may influence the likelihood of engagement with misinformation. Efforts to examine the impact of engagement with such misinformation on smokers’ beliefs and attitudes of relative harms of e-cigarettes and intentions to use e-cigarettes to reduce harm are needed, as a previous study has found that youth exposure to misinformation on YouTube can influence attitudes toward tobacco products [37]. The influence of the internet on population health is continuing to expand, and there is a need to better understand how people are increasingly engaging with “health social media” [18,38,39]. Evidence from this study is critical to inform future corrective interventions to address misperceptions and provide accurate information to smokers about relative harms of e-cigarettes [40,41]. Tools to mitigate misinformation, which have been used in other areas of public health that could be applied to e-cigarette–related information and misinformation may be through accuracy nudges, impactful hashtags, and web-based health communities [42-44]. Our findings can inform how information spreads on social media, and how future public health efforts and interventions can better understand likelihood of engagement on social media to combat the misinformation that exists on the internet.

## Appendix

Multimedia Appendix 1 Message stimuli with each condition.

Multimedia Appendix 2 Twitter engagement definitions provided in survey.

Multimedia Appendix 3 Descriptive Table of Mean (Standard Deviation) Values of 4 Engagement Behaviors by Condition.

Multimedia Appendix 4 Predictors of Likelihood of Engagement of Tweets among Twitter Users with Control Condition as Referent Group.

Multimedia Appendix 5 Predictors of Likelihood of Engagement of Tweets among Other Social Media Users with Control Condition as Referent Group.

Multimedia Appendix 6 Predictors of Likelihood of Engagement of Tweets Among Twitter users.

Multimedia Appendix 7 Predictors of Likelihood of Engagement of Tweets Among Other Social Media Users.

Multimedia Appendix 8 CONSORT-eHEALTH checklist (V 1.6.1).

## Abbreviations

CONSORT: Consolidated Standards of Reporting Trials
FDA: Food and Drug Administration
KR-20: Kuder-Richardson coefficient
NIH: National Institutes of Health
