# Supplementary material for: Smokers’ Likelihood to Engage With Information and Misinformation on Twitter About the Relative Harms of e-Cigarette Use: Results From a Randomized Controlled Trial
Source: JMIR Public Health Surveill. 2021 Dec 21;7(12):e27183. doi: 10.2196/27183 (PMC8734921; doi:10.2196/27183)
Supplement: Multimedia Appendix 2 [file publichealth_v7i12e27183_app2.pdf]

Appendix 2

Twitter engagement definitions provided in survey

“A reply is a response to another person’s Tweet, a Retweet is a re-posting of a Tweet, Likes are used to show appreciation for a Tweet, and you can Share a tweet via direct message, text message, or email.

What does reply, retweet, like, or share mean?

https://help.twitter.com/en/using-twitter/retweet-faqs”
