# Supplementary material for: Smokers’ Likelihood to Engage With Information and Misinformation on Twitter About the Relative Harms of e-Cigarette Use: Results From a Randomized Controlled Trial
Source: JMIR Public Health Surveill. 2021 Dec 21;7(12):e27183. doi: 10.2196/27183 (PMC8734921; doi:10.2196/27183)
Supplement: Multimedia Appendix 3 [file publichealth_v7i12e27183_app3.pdf]

Appendix 3. Descriptive Table of Mean (Standard Deviation) Values of 4 Engagement Behaviors by Condition

	Condition 1	Condition 2	Condition 3	Condition 4
Reply	0.62 (1.27)	0.68 (1.26)	0.59 (1.20)	0.63 (1.26)
Retweet	0.72 (1.36)	0.40 (0.98)	0.65 (1.20)	0.95 (1.43)
Like	1.38 (1.70)	0.64 (1.17)	1.17 (1.41)	2.04 (1.71)
Share	1.08 (1.60)	0.52 (1.11)	0.83 (1.31)	1.24 (1.57)

*Note: Each type of engagement had a score range of 0-4 (the sum of whether or not they would engage with each image)
