# Supplementary material for: Smokers’ Likelihood to Engage With Information and Misinformation on Twitter About the Relative Harms of e-Cigarette Use: Results From a Randomized Controlled Trial
Source: JMIR Public Health Surveill. 2021 Dec 21;7(12):e27183. doi: 10.2196/27183 (PMC8734921; doi:10.2196/27183)
Supplement: Multimedia Appendix 5 [file publichealth_v7i12e27183_app5.pdf]

Appendix 5. Predictors of Likelihood of Engagement of Tweets among Other Social Media Users with Control Condition as Referent Group.

Variables		Full (n=1287)				US (n=676)				UK (n=611)			
		Beta	95% CI (lower)	95% CI (higher)	P-value	Beta	95% CI (lower)	95% CI (higher)	P-value	Beta	95% CI (lower)	95% CI (higher)	P-value
Unadjusted													
Condition	Control (referent)												
	As or more harmful	-0.194	-0.270	-0.119	<0.001	-0.266	-0.365	-0.168	<0.001	-0.124	-0.242	-0.060	0.039
	Completely harmless	-0.880	-0.978	-0.784	<0.001	-0.907	-1.030	-0.785	<0.001	-0.906	-1.068	-0.747	<0.001
	Uncertainty	-0.449	-0.534	-0.364	<0.001	-0.563	-0.676	-0.451	<0.001	-0.321	-0.453	-0.190	<0.001
		Nagelkerke Pseudo-R^2 = 0.257				Nagelkerke Pseudo-R^2 = 0.314				Nagelkerke Pseudo-R^2 = 0.220			
		AIC = 9523.7				AIC = 5238.5				AIC = 4190.8			
Adjusted													
Condition	As or more harmful	0.482	0.396	0.568	<0.001	0.614	0.502	0.728	<0.001	0.567	0.395	0.742	<0.001
	Completely harmless	0.309	0.222	0.395	<0.001	0.375	0.261	0.490	<0.001	0.860	0.700	1.024	<0.001
	Uncertainty	-0.419	-0.525	-0.313	<0.001	-0.326	-0.462	-0.191	<0.001	0.761	0.600	0.925	<0.001
Country	US	-0.342	-0.411	-0.273	<0.001								
		-0.005	-0.008	-0.002	<0.001	-0.004	-0.007	0.000	0.035	-0.008	-0.012	-0.004	<0.001
Sex	Male	-0.085	-0.148	-0.022	0.008	-0.043	-0.127	0.041	0.314	-0.098	-0.196	0.000	0.049
Race	White	-0.199	-0.274	-0.123	<0.001	-0.308	-0.392	-0.223	<0.001	0.346	0.142	0.562	0.001
Education	Some college/ further education college	-0.007	-0.077	0.062	0.839	-0.029	-0.122	0.064	0.538	0.049	-0.058	0.156	0.371
	College/ University degree or higher	-0.136	-0.220	-0.052	0.002	-0.133	-0.239	-0.028	0.014	-0.100	-0.247	0.043	0.175
E-cigarette Use	Past e-cigarette Use	-0.052	-0.116	0.011	0.108	-0.049	-0.132	0.034	0.249	-0.030	-0.130	0.070	0.557
Social Media Use		0.177	0.149	0.204	<0.001	0.207	0.171	0.243	<0.001	0.144	0.103	0.186	<0.001
Daily Internet Use		0.013	0.006	0.020	<0.001	0.013	0.004	0.021	0.004	0.014	0.001	0.027	0.032
Baseline Perceived Relative Harm of E-cigarettes		0.023	-0.002	0.047	0.067	0.073	0.042	0.105	<0.001	-0.058	-0.098	-0.018	0.005
		Nagelkerke Pseudo-R^2 = 0.504				Nagelkerke Pseudo-R^2 = 0.589				Nagelkerke Pseudo-R^2 = 0.401			
		AIC = 9024.5				AIC = 4911.1				AIC = 4047.9			

*Note: Significant predictors are italicized
