# Supplementary material for: Smokers’ Likelihood to Engage With Information and Misinformation on Twitter About the Relative Harms of e-Cigarette Use: Results From a Randomized Controlled Trial
Source: JMIR Public Health Surveill. 2021 Dec 21;7(12):e27183. doi: 10.2196/27183 (PMC8734921; doi:10.2196/27183)
Supplement: Multimedia Appendix 6 [file publichealth_v7i12e27183_app6.pdf]

Appendix 6. Predictors of Likelihood of Engagement of Tweets Among Twitter users

Variables		Full (n=974)				US (n=449)				UK (n=525)			
		Beta	95% CI (lower)	95% CI (higher)	P-value	Beta	95% CI (lower)	95% CI (higher)	P-value	Beta	95% CI (lower)	95% CI (higher)	P-value
Unadjusted													
Condition	As or more harmful (referent)												
	Completely harmless	*-0.419*	*-0.516*	*-0.323*	*<0.001*	*-0.428*	*-0.559*	*-0.299*	*<0.001*	*-0.393*	*-0.537*	*-0.250*	*<0.001*
	Uncertainty	-0.038	-0.125	0.050	0.400	*-0.315*	*-0.440*	*-0.191*	*<0.001*	*0.234*	*0.110*	*0.360*	*<0.001*
	Control	*0.317*	*0.235*	*0.398*	*<0.001*	*0.175*	*0.067*	*0.285*	*0.002*	*0.452*	*0.330*	*0.575*	*<0.001*
		Nagelkerke Pseudo-R^2 = 0.251				Nagelkerke Pseudo-R^2 = 0.253				Nagelkerke Pseudo-R^2 = 0.296			
		AIC = 7560.4				AIC = 3687				AIC = 3784.4			
Adjusted													
Condition	Completely harmless	*-0.439*	*-0.536*	*-0.343*	*<0.001*	*-0.446*	*-0.578*	*-0.316*	*<0.001*	*-0.415*	*-0.559*	*-0.271*	*<0.001*
	Uncertainty	-0.069	-0.157	0.019	0.125	*-0.340*	*-0.466*	*-0.215*	*<0.001*	*0.202*	*0.077*	*0.328*	*0.002*
	Control	*0.300*	*0.218*	*0.382*	*<0.001*	*0.190*	*0.080*	*0.300*	*0.001*	*0.440*	*0.317*	*0.564*	*<0.001*
Country	US	*-0.207*	*-0.277*	*-0.137*	*<0.001*								
Age		*-0.004*	*-0.006*	*-0.001*	*0.002*	-0.003	-0.007	0.000	0.078	*-0.004*	*-0.008*	*0.000*	*0.027*
Sex	Male	0.046	-0.016	0.107	0.148	0.045	-0.042	0.133	0.306	0.028	-0.063	0.119	0.549
Race	White	*-0.289*	*-0.367*	*-0.211*	*<0.001*	*-0.292*	*-0.382*	*-0.201*	*<0.001*	*-0.275*	*-0.438*	*-0.106*	*0.001*
Education	Some college/ further education college	-0.030	-0.104	0.043	0.419	-0.039	-0.145	0.068	0.475	0.036	-0.070	0.142	0.507
	College/ University degree or higher	*-0.105*	*-0.184*	*-0.026*	*0.009*	*-0.131*	*-0.241*	*-0.020*	*0.021*	-0.076	-0.192	0.041	0.203
E-cigarette Use	Past e-cigarette Use	*-0.076*	*-0.138*	*-0.014*	*0.016*	*-0.083*	*-0.169*	*0.003*	*0.059*	-0.024	-0.115	0.067	0.607
Social Media Use		*0.135*	*0.118*	*0.153*	*<0.001*	*0.193*	*0.170*	*0.217*	*<0.001*	*0.062*	*0.035*	*0.088*	*<0.001*
Daily Internet Use		0.005	-0.002	0.011	0.183	-0.001	-0.010	0.008	0.882	*0.015*	*0.004*	*0.025*	*0.006*
Baseline Perceived Relative Harm of E-cigarettes		*0.037*	*0.012*	*0.061*	*0.003*	*-0.003*	*-0.040*	*0.033*	*0.853*	*0.073*	*0.038*	*0.106*	*<0.001*
		Nagelkerke Pseudo-R^2 = 0.532			0.079	Nagelkerke Pseudo-R^2 = 0.676				Nagelkerke Pseudo-R^2 = 0.411			
		AIC = 7122.4				AIC = 3330.5				AIC = 3708.6			

Note. Significant predictors are italicized.
