# Supplementary material for: Smokers’ Likelihood to Engage With Information and Misinformation on Twitter About the Relative Harms of e-Cigarette Use: Results From a Randomized Controlled Trial
Source: JMIR Public Health Surveill. 2021 Dec 21;7(12):e27183. doi: 10.2196/27183 (PMC8734921; doi:10.2196/27183)
Supplement: Multimedia Appendix 7 [file publichealth_v7i12e27183_app7.pdf]

Appendix 7: Predictors of Likelihood of Engagement of Tweets Among Other Social Media Users

Variables		Full (n=1287)				US (n=676)				UK (n=611)			
		Beta	95% CI (lower)	95% CI (higher)	P-value	Beta	95% CI (lower)	95% CI (higher)	P-value	Beta	95% CI (lower)	95% CI (higher)	P-value
Unadjusted													
Condition	As or more harmful (referent)												
	Completely harmless	*-0.686*	*-0.784*	*-0.588*	*<0.001*	*-0.641*	*-0.765*	*-0.518*	*<0.001*	*-0.781*	*-0.946*	*-0.622*	*<0.001*
	Uncertainty	*-0.254*	*-0.341*	*-0.168*	*<0.001*	*-0.297*	*-0.410*	*-0.184*	*<0.001*	*-0.197*	*-0.331*	*-0.065*	*0.004*
	Control	*0.194*	*0.119*	*0.270*	*<0.001*	*0.266*	*0.168*	*.0.365*	*<0.001*	*0.124*	*0.006*	*0.242*	*0.039*
		Nagelkerke Pseudo-R^2 = 0.257				Nagelkerke Pseudo-R^2 = 0.314				Nagelkerke Pseudo-R^2 = 0.220			
		AIC = 9523.7				AIC = 5238.5				AIC = 4190.8			
Adjusted													
Condition	Completely harmless	*-0.727*	*-0.826*	*-0.630*	*<0.001*	*-0.701*	*-0.826*	*-0.578*	*<0.001*	*-0.761*	*-0.925*	*-0.600*	*<0.001*
	Uncertainty	*-0.309*	*-0.395*	*-0.222*	*<0.001*	*-0.375*	*-0.490*	*-0.261*	*<0.001*	*-0.194*	*-0.328*	*-0.060*	*0.005*
	Control	*0.173*	*0.097*	*0.249*	*<0.001*	*0.239*	*0.140*	*0.338*	*<0.001*	0.099	-0.019	0.218	0.101
Country	US	*-0.342*	*-0.411*	*-0.273*	*<0.001*								
Age		*-0.005*	*-0.008*	*-0.002*	*<0.001*	*-0.004*	*-0.007*	*0.000*	*0.035*	*-0.008*	*-0.012*	*-0.004*	*<0.001*
Sex	Male	*-0.085*	*-0.148*	*-0.022*	*0.008*	-0.043	-0.127	0.041	0.314	*-0.098*	*-0.196*	*0.000*	*0.049*
Race	White	*-0.199*	*-0.274*	*-0.123*	*<0.001*	*-0.308*	*-0.392*	*-0.223*	*<0.001*	*0.346*	*0.142*	*0.562*	*0.001*
Education	Some college/ further education college	-0.007	-0.077	0.062	0.839	-0.029	-0.122	0.064	0.538	0.049	-0.058	0.156	0.371
	College/ University degree or higher	*-0.136*	*-0.220*	*-0.052*	*0.002*	*-0.133*	*-0.239*	*-0.028*	*0.014*	-0.100	-0.247	0.043	0.175
E-cigarette Use	Past e-cigarette Use	-0.052	-0.116	0.011	0.108	-0.049	-0.132	0.034	0.249	-0.030	-0.130	0.070	0.557
Social Media Use		*0.177*	*0.149*	*0.204*	*<0.001*	*0.207*	*0.171*	*0.243*	*<0.001*	*0.144*	*0.103*	*0.186*	*<0.001*
Daily Internet Use		*0.013*	*0.006*	*0.020*	*<0.001*	*0.013*	*0.004*	*0.021*	*0.004*	*0.014*	*0.001*	*0.027*	*0.032*
Baseline Perceived Relative Harm of E-cigarettes		0.023	-0.002	0.047	0.067	*0.073*	*0.042*	*0.105*	*<0.001*	*-0.058*	*-0.098*	*-0.018*	*0.005*
		Nagelkerke Pseudo-R^2 = 0.504				Nagelkerke Pseudo-R^2 = 0.589				Nagelkerke Pseudo-R^2 = 0.401			
		AIC = 9024.5				AIC = 4911.1				AIC = 4047.9			

Note. Significant predictors are italicized.
